# Sonic Hedgehog Medulloblastoma Cancer Stem Cells Mirnome and Transcriptome Highlight Novel Functional Networks

**DOI:** 10.3390/ijms19082326

**Published:** 2018-08-08

**Authors:** Agnese Po, Luana Abballe, Claudia Sabato, Francesca Gianno, Martina Chiacchiarini, Giuseppina Catanzaro, Enrico De Smaele, Felice Giangaspero, Elisabetta Ferretti, Evelina Miele, Zein Mersini Besharat

**Affiliations:** 1 Department of Molecular Medicine, Sapienza University, 00161 Rome, Italy; agnese.po@uniroma1.it (A.P.); claudia.sabato@uniroma1.it (C.S.); martina.chiacchiarini@uniroma1.it (M.C.); 2 Department of Experimental Medicine, Sapienza University, 00161 Rome, Italy; luana.abballe@uniroma1.it (L.A.); giuseppina.catanzaro@uniroma1.it (G.C.); enrico.desmaele@uniroma1.it (E.D.S.); zeinmersini.besharat@uniroma1.it (Z.M.B.); 3 Center for Life Nano Science@Sapienza, Istituto Itialiano di Tecnologia, 00161 Rome, Italy; evelina.miele@opbg.net; 4 Department of Radiological, Oncological and Pathological Science, Sapienza University, 00161 Rome, Italy; francesca.gianno@uniroma1.it (Fr.G.); felice.giangaspero@uniroma1.it (Fe.G); 5 IRCCS Neuromed, Pozzilli, 86077 Isernia, Italy; 6 Department of Hematology/Oncology and Stem Cell Transplantation, Bambino Gesù Children’s Hospital, Istituto di Ricovero e Cura a Carattere Scientifico, 00165 Rome, Italy

**Keywords:** microRNAs, cancer stem cells, medulloblastoma, Sonic Hedgehog pathway, RNA-sequencing

## Abstract

Molecular classification has improved the knowledge of medulloblastoma (MB), the most common malignant brain tumour in children, however current treatments cause severe side effects in patients. Cancer stem cells (CSCs) have been described in MB and represent a sub population characterised by self-renewal and the ability to generate tumour cells, thus representing the reservoir of the tumour. To investigate molecular pathways that characterise this sub population, we isolated CSCs from Sonic Hedgehog Medulloblastoma (SHH MB) arisen in Patched 1 (*Ptch1*) heterozygous mice, and performed miRNA- and mRNA-sequencing. Comparison of the miRNA-sequencing of SHH MB CSCs with that obtained from cerebellar Neural Stem Cells (NSCs), allowed us to obtain a SHH MB CSC miRNA differential signature. Pathway enrichment analysis in SHH MB CSCs mirnome and transcriptome was performed and revealed a series of enriched pathways. We focused on the putative targets of the SHH MB CSC miRNAs that were involved in the enriched pathways of interest, namely pathways in cancer, PI3k-Akt pathway and protein processing in endoplasmic reticulum pathway. In silico analysis was performed in SHH MB patients and identified several genes, whose expression was associated with worse overall survival of SHH MB patients. This study provides novel candidates whose functional role should be further investigated in SHH MB.

## 1. Introduction

Medulloblastoma (MB) is the most common malignant brain tumour in childhood. Multimodal aggressive treatments include surgical resection and chemo- and radio-therapy, and are able to cure about 70% of patients. Unfortunately, due to these cytotoxic and aggressive therapies, survivors often face severe side effects. Moreover, one third of patients still die of disease due to relapse and metastasis. Recent years have witnessed an intense molecular characterisation of MBs, according to the genetic structure, gene expression pattern, and methylation profile [1,2,3,4,5] with the final aim to correlate molecular features to risk categories, a better management of MB patients, and the identification of molecular targets and molecular pathways to be exploited as new therapeutic strategies. Despite being initially characterised according to histological features, the 2016 WHO consensus conference envisaged five subgroups: Wingless-type MMTV integration site family (WNT) activated, Sonic Hedgehog (SHH) activated P53 wild-type, SHH activated P53 mutant, non-WNT/non-SHH Group 3, non-WNT/non-SHH Group 4 [6]. Research groups continue implementing and integrating molecular and clinical features, and recent reports described up to 12 molecular subtypes that more deeply characterise the 4 subgroups that were originally described by Northcott [4]: WNT has 2 subtypes (α and β), SHH has 4 subtypes (α, β, γ and δ), group 3 and 4 have 3 subtypes each (α, β and γ) [2].

Among MB molecular subgroups, SHH subgroups (P53 wild-type and mutant) according to WHO, α, β, γ, and δ according to Cavalli et al. [2] account for roughly 24–30% of cases. Attempts were carried out recently to identify possible therapeutic targets, with particular efforts to find drugs targeting Smoothened (SMO) [7,8], the upstream regulator of the pathway. However, despite good results in other SHH driven cancers, such as basal cell carcinoma [9,10,11], in SHH MB the initial response was followed by resistance and progression of disease [12,13]. As a result, the crosstalk of SHH pathway with other pathways [14] should be taken into account in the design and development of novel drugs since tumour escape has been reported. Recently, two novel SMO inhibitors, based on Vismodegib (GDC-0449), decreased *Ptch*^+/−^/*p53*^−/−^ MB allograft tumour growth [15], however further investigations are required before a possible clinical trial.

Cancer stem cells (CSCs) may arise from the malignant transformation of neural stem cells (NSCs) of more committed progenitors [16] and have been described from MB [17]. There are several SHH based MB mouse models [18]. Goodrich et al. [19] established a model based on the mutation of *Ptch*, the transmembrane receptor of SHH that also functions as a tumor suppressor and reported that a subset of *Ptch* heterozygotes spontaneously develops MB. We previously isolated CSCs from human and murine *Ptch* heterozygous SHH MB [20,21,22,23] and we demonstrated that they share common features with NSCs derived from postnatal cerebellum [20]. Indeed, SHH MB CSCs and NSCs are both sustained by the Hedgehog (HH) signaling pathway using the glioma-associated oncogene homolog to up-regulate the stemness factor *Nanog* [20]. Moreover, we showed that *NFκB* (Nuclear factor Kappa B DNA binding subunit), a transcription regulator, has a role in the sustenance of human SHH MB CSCs [21]. Interestingly, we identified several upstream regulators of *NFκB* [21] such as *NPM* (Nucleophosmin), *PCNA* (Proliferating Nuclear Antigen), *P65* (Nuclear Factor Kappa B Transcription Factor P65), and *HSPA1A* (Heat Shock Protein Family A [Hsp70] Member 1A) potentially involved in the induction of aberrant cell growth and proliferation in human SHH MB CSCs through the combined analysis of proteomics and microRNA (miRNA) expression profiles [22]. The importance of HH–GLI signaling in tumour maintenance and stemness properties [24] has been also described in other CSC contexts, such as colon and lung cancer, in various ways for instance through a non-canonical signalling, involving *NRP2* (Neuropilin 2) and MAP/ERK (Mitogen Activated Protein Kinase/Extracellular Signal-Regulated Kinase) signalling [25].

Since CSCs are considered to be the ultimate reservoir of cancer and responsible for relapse and metastasis, it is thus of great interest to investigate molecular mechanisms represented at high levels in these cells.

## 2. Results and Discussion

### 2.1. miRNA-Sequencing Determines miRNA Signatures in SHH MB CSCs

**Figure 1:**
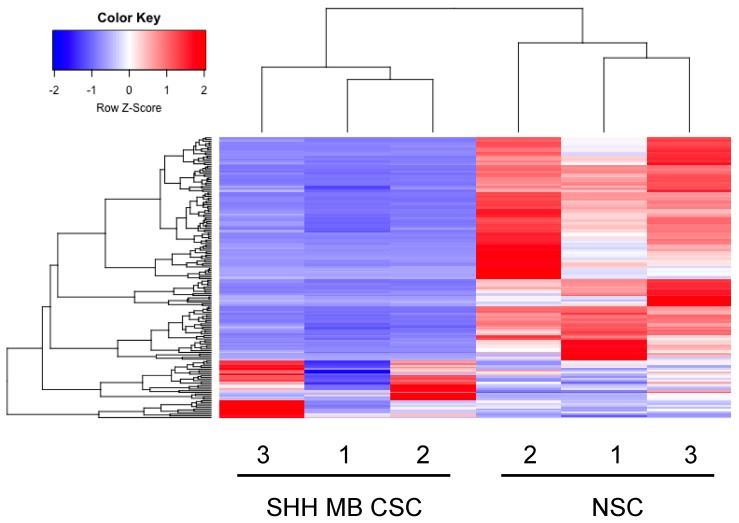
miRNA-sequencing determines miRNA signatures in Sonic Hedgehog Medulloblastoma Cancer Stem Cells (SHH MB CSCs). Heat map and dendrogram depiction of the 168 miRNAs displaying significant differential expression in SHH MB CSCs and Neural Stem Cells (NSCs).

**Figure 2:**
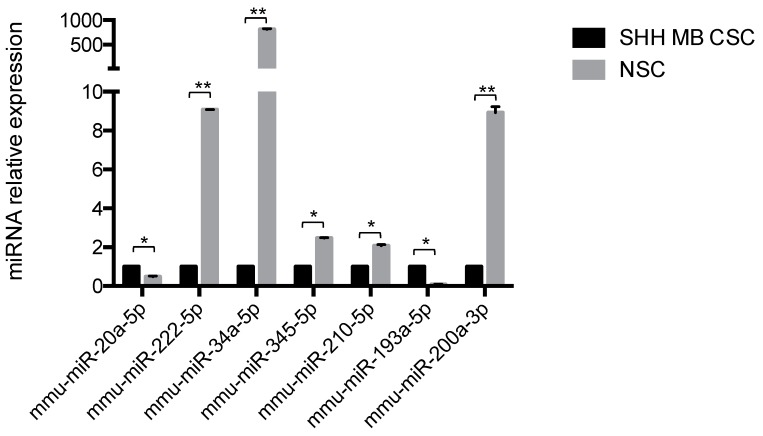
Quantitative PCR validation of mirnome in SHH MB CSC versus NSC. miRNA expression level of miRNAs (miR-20a-5p, miR-222-5p, miR-34a-5p, miR-345-5p, miR-210-5p, miR-193a-5p, and miR-200a-3p) in SHH MB CSCs vs. NSCs. Data are normalised versus endogenous control: U6. Results are shown in relative expression and data are means ± SD from three independent experiments. *p*-values * *p* < 0.05; ** *p* < 0.01.

SHH MB CSCs were subjected to small RNA sequencing and miRNA expression levels were compared to our recently published NSC mirnome [26]. Hierarchical clustering of the differentially expressed miRNAs yielded two distinct clusters, clearly distinguishing SHH MB CSCs and NSCs (Figure 1). In detail, SHH MB CSCs were characterised by 35 up-regulated and 133 down-regulated miRNAs (Table 1 and Table 2 report the top 20 miRNAs, respectively; the entire datasets are in Tables S1 and S2). Results of the miRNA-sequencing were validated at the transcriptional level by quantitative PCR. Higher expression of miR-20a-5p and miR-193a-5p and lower expression of miR-222-5p, miR-34a-5p, miR-345-5p, miR-210-5p, and miR-200a-3p were confirmed in SHH MB CSCs compared to NSCs (marked in bold in Tables S1 and S2, Figure 2). The SHH MB CSC miRNA signature included several miRNAs that have been described in primary MBs [27,28,29,30]. Specifically, among miRNAs characterising SHH MB CSCs, we identified let-7a, miR-100, miR-132, miR-135a, miR-135b, miR-150, and miR-203, which we had previously described as deregulated miRNAs in primary human SHH MBs (originally named Gli^high^) in respect to non-SHH MBs (Gli^low^) [27]. Moreover, miR-135b and miR-203 were similarly down-regulated as in Gli^high^ in respect to Gli^low^ MBs, possibly representing the MB CSC compartment. Northcott et al. [30] performed a comparison between MB subgroups and identified a number of SHH MB-related miRNAs, several of which were deregulated in SHH MB CSCs, such as miR-20a, miR-17, miR-135a, miR-106a, miR-222, miR-135b, and miR-203. Of particular interest are the up-regulated miRNAs (miR-20a and miR-17) and the down-regulated miRNAs (miR-135b and miR-203) that were similarly expressed in both studies. Moreover, in the same study of Northcott et al. the expression of miRNAs in Shh-treated cerebellar granule neuron precursors (CGNPs) is reported and miR-19b, miR-714, and miR-709 showed a coherent deregulation with SHH MB CSCs. We previously reported miRNAs expressed in primary MBs compared to normal adult and fetal human cerebellum [28]. Interestingly, thirteen miRNAs deregulated in primary MB were also deregulated in SHH MB CSCs (miR-203, miR-361, miR-31, miR-17, miR-20a, miR-106a, miR-let-7a, miR-132, miR-150, miR-212, miR-330, miR-29a, and miR-135a) and of particular interest miR-203, miR-361, miR-31, miR-17, and miR-20a were expressed in a similar fashion in both primary MBs and SHH MB CSCs. Further studies have been performed focusing on miRNA expression in MB mouse models and as a result the collaboration of miR-17~92 cluster with the HH pathway in MB and its up-regulation in SHH MB [29,31].

### 2.2. Enriched Pathway Analysis of SHH MB CSCs Transcriptome and Mirnome

**Figure 3:**
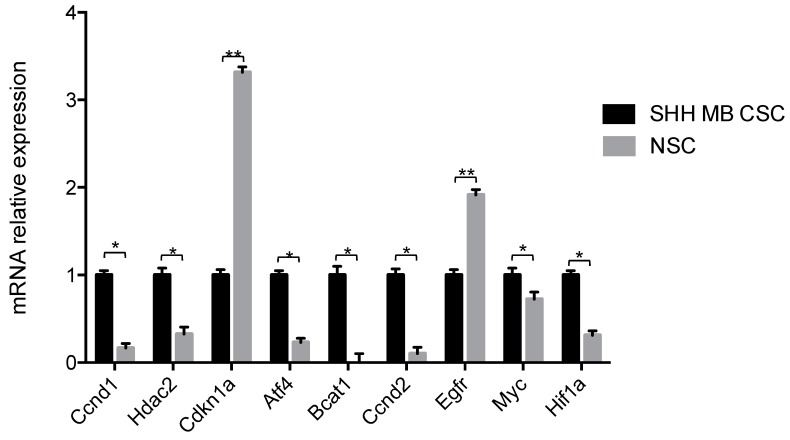
Validation of transcriptome in SHH MB CSC versus NSC. mRNA expression levels of *Ccnd1*, *Hdac2*, *Cdkn1a*, *Atf4*, *Bcat1*, *Ccnd2*, *Egfr*, *Myc*, and *Hif1a* in SHH MB CSCs compared to NSCs. Results are shown in relative expression and data are means ± SD from three independent experiments. *p*-values *p < 0.05; ** *p* < 0.01.

**Figure 4:**
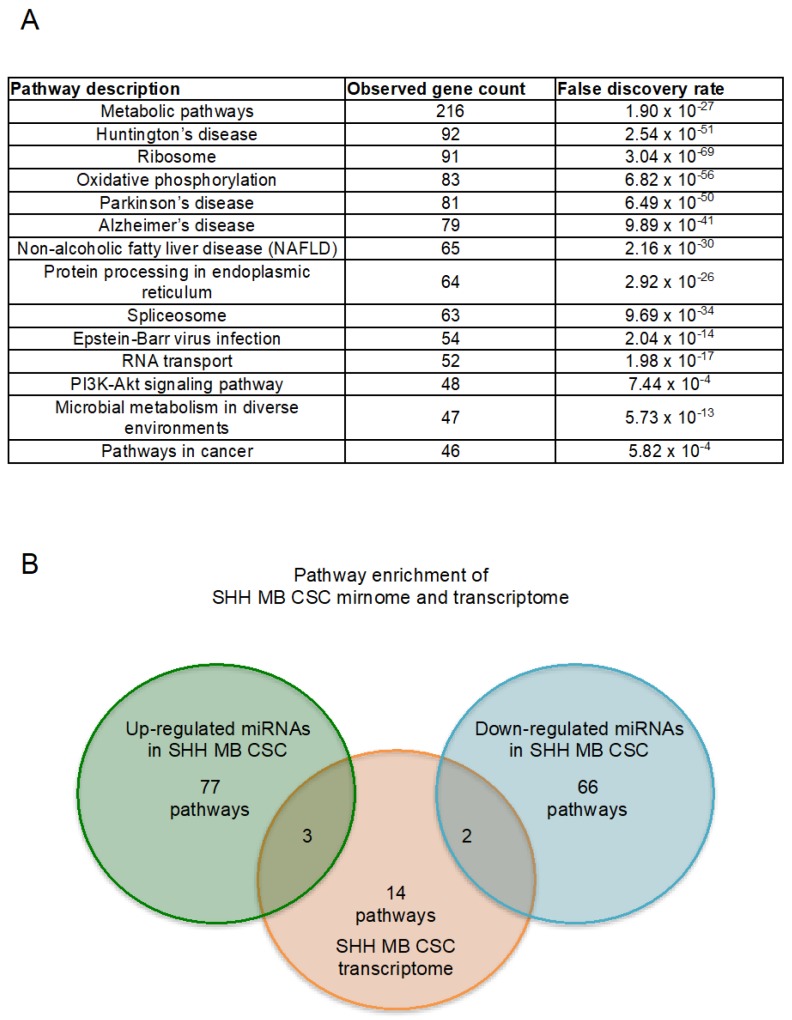
Enriched pathway analysis of SHH MB CSC transcriptome and mirnome. (**A**) Enriched pathways over-represented in the set of genes obtained through mRNA-sequencing of SHH MB CSCs *Findings are ranked according to observed gene count; (**B**) Venn diagram reporting the enriched pathways identified in SHH MB CSC up-regulated miRNAs, transcriptome and down-regulated miRNAs. (The enriched pathways identified in all three SHH MB CSC contexts are shown in Table 3, Table 4 and Table 5 and Tables S4–S6, respectively).

SHH MB CSCs were next subjected to mRNA-sequencing and transcripts were ranked according to their expression (Table S3). mRNA-sequencing results were validated at the transcriptional level by quantitative PCR. Higher expression of *Ccnd1*, *Hdac2*, *Atf4*, *Bcat1*, *Ccnd2*, *Myc* and *Hiflf1a* was confirmed in SHH MB CSCs, whereas higher expression of *Cdkn1a* and *Egfr* was confirmed in NSCs (Figure 3). In order to determine the enriched pathways of SHH MB CSCs, pathway enrichment analysis was performed. The statistically significant pathways with the highest gene count, and thus most affected, are reported in Figure 4A. Among them, the decrease on oxidative phosphorylation has already been described in MB and the combinatorial targeting of MB metabolism has been proposed for effective treatments [32]. Of equal interest is the protein processing in endoplasmic reticulum pathway since endoplasmic reticulum stress has been reported to promote angiogenesis, invasiveness, and MB tumour growth in a murine model [33]. Moreover, the enrichment of PI3K-Akt signalling pathway and pathways in cancer were identified. The PI3K-Akt/mTOR pathway has been reported to be activated in MB [34,35,36], involved in metastasis [37] and its targeting has been proposed to inhibit MB CSCs [38] and overcome resistance to SMO antagonists [39]. All enriched pathways along with their matching genes are listed in Table 3 and Table S4, findings are ranked according to observed gene count.

Accordingly, pathway enrichment analysis was performed for the SHH MB CSC miRNAs. In detail, up-regulated and down-regulated miRNAs were used as inputs and 25 pathways were reported as statistically significant for each input (Table 4 and Table 5 report the top 20 pathways. The entire tables are reported in Tables S5 and S6). Since we were interested in the identification of the pathways characterising SHH MB CSCs, we focused on the intersection of the enriched pathways between the SHH MB CSC miRNAs and their transcriptome. Pathways in cancer, PI3K-Akt pathway, and protein processing in endoplasmic reticulum were the three common pathways identified by the intersection between the enriched pathways of the up-regulated miRNAs and those of the SHH MB CSC transcriptome (Figure 4B). Whereas, only two pathways were in common between the down-regulated miRNAs in SHH MB CSCs and their transcriptome, as shown in Figure 4B, pathways in cancer and PI3K-Akt pathway.

### 2.3. Novel Functional Networks in SHH MB CSCs

**Figure 5:**
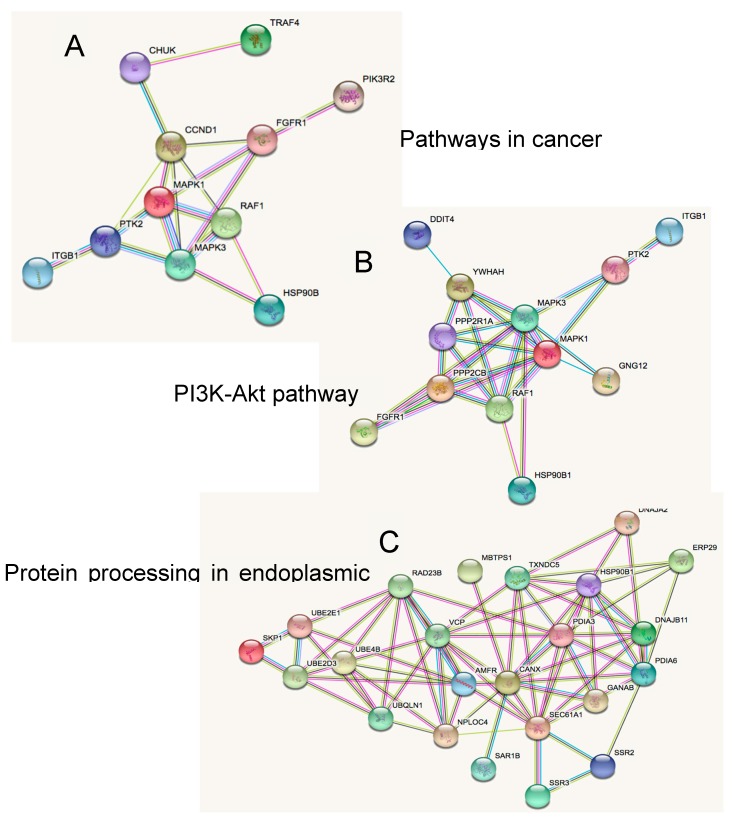
Putative targets of up-regulated miRNAs in SHH MB CSC enriched pathways, include (**A**) pathways in cancer, (**B**) PI3K-Akt pathway, and (**C**) protein processing in endoplasmic reticulum.

The binding of miRNA to mRNA is a type of regulation that can control both the translation of the mRNA and the availability of miRNA for other targets [40], indeed miRNAs post-transcriptional regulation can amount to controlling 50% of the protein-coding genes, due to the ability of miRNAs to target multiple genes [41]. On this basis, we focused on the identification of networks that could be involved in the perpetuation of SHH MB CSCs using the putative targets of the up-regulated and down-regulated miRNAs that belong to the common enriched pathways. As reported in Figure 5, we firstly focused on the networks comprising the putative targets of the up-regulated miRNAs. Networks in Figure 5A,B show several genes in common, such as *Mapk1*, *Mapk3*, *Raf1*, *Fgfr1*, *Ptk2*, and *Itgb1*. All of them play a pivotal role in cancer cell growth and maintenance. Specifically, *Mapk1* and *Mapk3* activation have been associated with metastatic MB and poor outcomes [42]. Also, resistance to SMO inhibition along with metastasis in SHH dependent tumours has been attributed to the activation of RAS/MAPK pathway [43]. *Fgfr1* is a member of the fibroblast growth receptors family and activation of Fgf signalling has been proposed as a target of MB and other SHH dependent tumours [44]. Similarly, *Ptk2*, a focal adhesion kinase, has been reported to induce MB cells proliferation and mediate c-Met migration and invasion [45]. Recently, another study investigated the activation of *Itgb1* by *Map4k4* and its association with the infiltration of MB cells [46]. Interestingly, *Hsp90b1* was involved in all three networks (Figure 5A–C). *Hsp90b1* is an essential molecular chaperone [47] and it has recently emerged that inhibitors of *Hsp90b1*, together with inhibitors of the other proteins of the Hsp90 family, are promising classes of anti-cancer drugs in both solid and hematologic malignancies [48].

**Figure 6:**
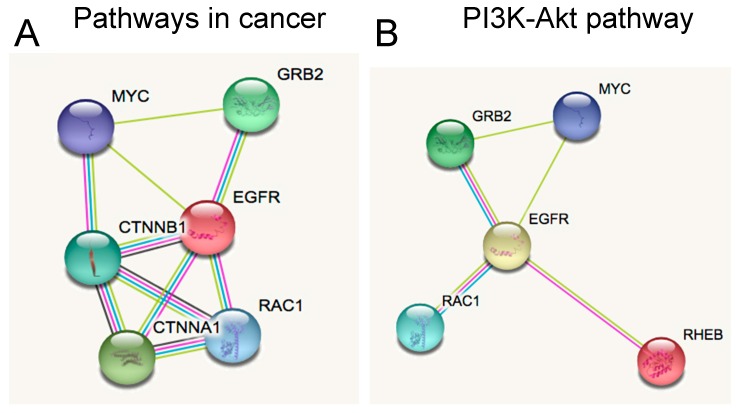
Putative targets of down-regulated miRNAs in SHH MB CSC enriched pathways, include (**A**) pathways in cancer and (**B**) PI3K-Akt pathway.

On the other hand, from the putative targets of the down-regulated miRNAs in SHH MB CSCs, we observed the genes involved in both networks and found some crucial genes involved in MB progression, including *Myc*, *Grb2*, *Egfr*, and *Rac1*, as shown in Figure 6A,B. In detail, *Myc* has been identified as a marker of tumour aggressiveness and SHH activation in the SHH MB subgroup [4,49,50]. Next, we found two components of the Egfr pathway, whose function consists of the adaptor molecule Grb2 binding to *Egfr* with the subsequent activation of the pathway. ERK activation has been associated with MB progression and control of cell-cycle related proteins’ translation, possibly through mTOR activation [51]. Moreover, SHH induced *Rac1* activation and migration of fibroblasts [52], a mechanism that was also reported in highly invasive MB cells [53].

MiRNAs have been associated not only with tumorigenesis but also as prognostic biomarkers with the first study performed by Calin et al. describing the association of loss of miR-15 and miR-16 expression with a more favourable prognosis in chronic lymphocytic leukemia patients [54]. Unfortunately, even though treatment of MB patients has improved, 30% of patients still die from the disease [55]. Extending our research, we questioned if any of the putative targets of the SHH MB CSC miRNAs could have a role in the overall survival of MB patients [2]. Following the description of Cavalli et al. [2] regarding the four subtypes of SHH MB (α, β, γ, δ) being enriched for different age groups, we decided to take into consideration the factor of age in overall survival. To this end, in silico analysis was performed and survival curves were estimated in association with each gene expression level in MB patients divided in age groups. Firstly, infant (under 3 years of age) and pediatric (between 4 and 17 years of age) SHH MB patients together, then infant SHH MB, pediatric SHH MB, and finally adult SHH MB patients. Gene expression was associated with overall survival (OS) for each category and detailed results are reported in Table 6. Interestingly, expression levels of several genes were associated with worse overall survival of SHH MB patients. In detail, worse overall survival in infant and pediatric SHH MB patients was reported with high expression of *RHEB* and *UBQLN1* and low expression of *CHUK*, *CTNNB1*, and *DDIT4*. *MBTPS1*, *RAF1*, *TXNDC5*, and *VCP* high expression and *SAR1B* and *UBE4B* low expression were associated with worse overall survival in infant SHH MB patients. Additionally, high expression of *PPP2R1A*, *RAD23B*, *RHEB*, and *UBQLN1* and low expression levels of *CHUK* and *CTNNB1* were observed in pediatric SHH MB patients with worse overall survival. The absence of common genes among infant SHH MB patients and the other two categories is noteworthy. Infant MB patient treatment is extremely challenging [56] and this singularity of infant SHH MB patients could provide useful information since only *RAF1* is part of the PI3K-Akt pathway, whereas *MBTPS*, *TXNDC5*, *VCP*, *SAR1B*, and *UBE4B* are part of the protein processing in endoplasmic reticulum pathway. On the other hand, in pediatric SHH MB patients, *RHEB* is involved in the PI3K-Akt pathway, *CHUK* and *CTNNB1* in pathways in cancer and *PPP2R1A*, *RAD23B*, and *UBQLN1* are part of the protein processing in endoplasmic reticulum pathway. Interestingly, two genes belonging in pathways in cancer, *EGF*R and *MAPK3*, were identified only in adult MB patients. Low expression of *EGFR* and high expression of *MAPK3* were associated with worse OS in adult MB patients. We further investigated the putative targets of SHH MB CSCs taking into consideration the four SHH subtypes, pediatric SHHα, infant SHHβ, infant SHHγ, and adult SHHδ, as described by Cavalli et al. [2]. The OS analysis was performed for all putative targets of SHH MB CSC miRNAs for each SHH subtype and the statistically significant results are reported in Table 7. In detail, worse OS was associated with *CTNNB1*, *PDIA3*, and *RAF1* low expression and *RAD23B*, *RHEB*, *UBQLN1*, and *YWHAH* high expression in pediatric SHHα patients. Notably, *CTNNB1*, *RAD23B*, *RHEB*, and *UBQLN1* were also reported in pediatric MB patients (Table 6). Infant SHHβ patients were associated with worse OS when low expression of *UBE4B* was observed and this gene was also reported in infant MB patients (Table 6). None of the putative targets of the SHH MB CSC miRNAs was associated with worse OS in infant SHHγ, a subtype characterised by a good prognosis. Finally, in adult SHHδ patients low expression of *CHUK* and *DNAJB11* were associated with worse OS, these genes were not reported in the analysis regarding adult MBs (Table 6).

Whether the genes of interest reported in Table 6 could be characteristic only of SHH MB patients led to further in silico analysis of the gene expression levels between WNT, SHH, Group 3, and Group 4 MB patients and the results are reported in Table 8. Interestingly, a statistically significant higher expression of *CTNNB1*, *DDIT4* was observed in infant and pediatric SHH MB patients, whose low expression was associated with worse OS. Moreover, in the same age group higher expression of *RHEB* was observed in Group 4 MB patients compared to other MB subgroups, whose high expression reported a worse OS. Low expression of *MBTPS1* was observed in infant SHH and WNT MB patients compared to Group 3 and Group 4 MB patients and its high expression was associated with a worse OS. When comparing only pediatric MB patients the high expression of *CTNNB1* was observed in SHH MB patients compared to the other subgroups and its low expression was linked to worse OS. Of note, lower expression of *RAD23B* and *UBQLN1* was observed in pediatric SHH MB when compared to other subgroups and their high expression was associated to worse overall survival. Moreover, in WNT pediatric patients low expression of *RHEB* was observed when compared to other subgroups and its high expression was reported in patients with worse OS. Finally, in adult SHH MB patients high expression of *EGFR* was observed when compared to other MB subgroups and its low expression was related with worse OS.

Recently, researchers have also focused their research in understanding the GLI crosstalk with other pathways in order to develop possible combinational treatments [57]. Specifically, EGFR and GLI crosstalk has been reported in MB [58], whereas in other tumour contexts such as anaplastic thyroid cancer, triple breast cancer, and colon cancer GLI crosstalk has been reported with RAS-BRAF-ERK [59], WNT [60], and WNT/β-catenin [61] signalling pathways, respectively.

Our results report changes in the enriched pathways and changes in the number of genes that participate in each pathway, shedding light on another aspect of SHH MBs and identifying novel molecules and pathways that should be taken into consideration in future studies.

## 3. Materials and Methods

Unless otherwise specified, all commercial products were used in accordance with the manufacturer’s protocol.

### 3.1. SHH MB CSCs and NSCs

Murine SHH MB CSCs were derived, as previously described, from spontaneous tumours arisen in Ptch1^+/−^ mice [20], maintained as in Reference [23] and RNA extraction was performed as in Reference [62]. NSC mirnome data were obtained from our recently published manuscript [26]. Animal experiments were performed according to the European Community Council Directive 2010/63/EU and were approved by the local Ethics Committee for Animal Experiments (Prot. N 03/2013, 12/March/2013) of the Sapienza University of Rome.

### 3.2. miRNA-Sequencing

Three biological replicates of SHH MB CSCs were subjected to miRNA-sequencing, quality control, mapping, quantification and differential expression analysis was performed between SHH MB CSCs and NSCs, as previously described [26]. In detail, an average of 10.48 ± 0.748 million reads per biological replicate were obtained by the sequencing and quality control was performed using the FastQC tool (http://www.bioinformatics.babraham.ac.uk/projects/fastqc/, accessed on 01/Februay/2017 before mapping where an average of 92% of reads with a quality score >30 were obtained. All sequenced single-end reads (31 bases) were aligned to the to the *Mus musculus* small RNA library (12-2013), (miRBase V20) using the Genomatix Mining Station (GMS, Sesame 2.4, https://www.genomatix.de/, accessed on 19/February/2017) resulting in an average of 79% mapping. Differential expression analysis was performed using the miRNA-seq reads of SHH MB CSCs vs NSCs in the Genomatix Genome Analyzer (GGA, v3.30126, https://www.genomatix.de/, accessed on 21/February/2017) using the DESeq2 method. Differences characterised by a minimum log2 fold change of 1 and an adjusted *p*-value of < 0.05 (Benjamini–Hochberg correction for multiple testing) were statistically significant.

### 3.3. mRNA-Sequencing

Three biological replicates of SHH MB CSCs were subjected to mRNA sequencing, quality control, mapping and transcript abundance were obtained using the TopHat/Cufflinks pipeline, as previously described [26]. In detail, an average of 80.91 ± 5.41 million reads per biological replicate were obtained by the sequencing and quality control was performed using the FastQC tool (http://www.bioinformatics.babraham.ac.uk/projects/fastqc/, accessed on 1 February 2017) before mapping where we obtained an average of 86% of reads with a quality score >30. All sequenced 70mer paired-end reads were aligned to the *Mus musculus* genome (GRCm38 mm10 ENSEMBL) using TopHat (version 2.0.11, John Hopkins University, Baltimore, MD, USA) and resulted in an average of 80% mapping. Transcript assembly was performed with Cufflinks (version 2.2.2, University of Washington, Seattle, WA, USA) and transcript abundance was estimated using fragments per kilobase of exon per million fragments mapped (FPKM) values resulting in an average of 14,410 transcripts per biological replicate.

### 3.4. RNA Extraction and Gene Expression Analysis

Total RNA was isolated from cells using Trireagent (Ambion, Carlsbad, CA, USA) and reverse transcribed in cDNA as previously described [26]. cDNA was analysed in quantitative RT-PCR (qRT-PCR) using ViiA™ 7 Real-Time PCR System (Applied Biosystems, Waltham, MA, USA) and SensiFAST™ Probe Lo-ROX (Bioline Reagents Limited, London, UK). For each sample 10 ng of cDNA were used in qPCR. We selected TaqMan gene expression assays from Applied Biosystems (Applied Biosystems, Waltham, MA, USA): Ccnd1 (Mm00432359_m1); Hdac2 (Mm00515108_m1); Cdkn1a (Mm04205640_g1); Atf4 (Mm00515325_g1); Bcat1 (Mm00500289_m1); Ccnd2 (Mm00438070_m1); Egfr (Mm01187858_m1); Myc (Mm00487804_m1); Hif1a (Mm00468869_m1). β-2-Microglobulin (Mm00437762_m1), Hprt (Mm03024075_m1), and Actb (Mm02619580_g1) were used as endogenous controls.

MiRNA expression was assessed with TaqMan probes (Applied Biosystems, Waltham, MA, USA), as previously described [27], using the following miRNAs: miR-20a (Code: 000580); miR-222-5p (Code: 463390_mat); miR-34a (Code: 000426); miR-345-5p (Code: 002528); miR-210-5p (Code: 462444_mat); miR-193a-5p (Code: 002577); miR-200a-3p (Code: 000502). U6 snRNA (Code: 001973) was used as endogenous control.

mRNA quantification was expressed in arbitrary units and each amplification reaction was performed in triplicate. All data were evaluated using the 2^-ΔΔCT^ method and values were normalised to three endogenous controls.

### 3.5. Pathway Enrichment Analysis

DAVID (https://david.ncifcrf.gov/, accessed on 27 February 2017) [63] was used to perform pathway enrichment analysis using the KEGG (Kyoto Encyclopedia of Genes and Genomes) database for all the transcripts reported from the mRNA-sequencing and pathways enriched for more than 45 genes that were deemed of interest. Up-regulated miRNAs in SHH MB CSCs were used as input to obtain the enriched pathways and the same analysis was performed for the down-regulated miRNAs in SHH MB CSCs. Pathways with FDR < 0.05 and *p* < 0.05 were statistically significant and are reported in detail in Table 3, Table 4 and Table 5 and Tables S4–S6, respectively.

### 3.6. miRNA Putative Targets

Identification of miRNA putative targets was performed with miRSystem (http://mirsystem.cgm.ntu.edu.tw/, accessed on 12 January 2018)) [64] and target predictions for up-regulated and down-regulated miRNAs in SHH MB CSCs were obtained. The predicted targets were intersected with the genes included in the enriched pathways for the up-regulated (Tables S7–S9) and down-regulated miRNAs (Tables S10 and S11).

### 3.7. Functional Networks

The construction of the functional networks was performed with String DB (https://string-db.org/, accessed on 30 January 2018)) [65]. The genes of interest reported in Tables S7–S9 for the up-regulated miRNAs and in Tables S10 and S11 for the down-regulated miRNAs in SHH MB CSCs were used as input after the exclusion of the common genes between the predicted targets of the up-regulated and down-regulated miRNAs of the same pathway. Medium confidence interaction score was selected and networks were exported from the database.

### 3.8. In Silico Analysis of SHH MB Patients’ Overall Survival

Gene expression levels of infant (0–3 years *n* = 50), pediatric (4–10 years *n* = 45; 10–17 years *n* = 26, total *n* = 71), adult (>18 years *n* = 49) SHH MB patients, and SHHα (*n* = 41), SHHβ (*n* = 21), SHHγ (*n* = 25), and SHHδ (*n* = 41) MB patients (GEO ID: gse85217) were queried in R2 platform (https://hgserver1.amc.nl/cgi-bin/r2/main.cgi, accessed on 12 March 2018 and 29 July 2018) [66], using the probes reported in Table 6 and Table 7, and Kaplan–Meier survival curves were plotted along with the statistical analysis and Bonferroni-adjusted *p*-values < 0.05 (Table 6 and Table 7) were considered as statistically significant. Gene expression levels of infant and pediatric MB patients (SHH *n* = 146, WNT *n* = 51, Group 3 *n* = 131, Group 4 *n* = 300), infant MB patients (SHH *n* = 62, WNT *n* = 1, Group 3 *n* = 24, Group 4 *n* = 11), pediatric MB patients (SHH *n* = 84, WNT *n* = 50, Group 3 *n* = 107, Group 4 *n* = 289), and adult MB patients (SHH *n* = 69, WNT *n* = 13, Group 3 *n* = 5, Group 4 *n* = 14) (GEO ID: gse85217) were queried in R2 platform and compared for each age group among the four MB subgroups (WNT, SHH, Group 3, and Group 4) using the probes reported in Table 8.

## 4. Conclusions

This study allowed us to focus on the CSC compartment of SHH MB and the data obtained from mirnome and transcriptome sequencing brought in evidence of the implicated pathways in SHH MB CSCs. The activation of PI3K-Akt pathway has been reported in MBs and new studies are focusing on the combinational treatment of MB with SMO and PI3K-Akt inhibitors. On this basis, this study presents other candidate pathways and genes whose inhibition or activation could be the foundation of future functional studies, leading to a shift in the landscape of survival in SHH MB patients.

## Figures and Tables

**Table 1:** Top 20 up-regulated miRNAs in Sonic Hedgehog Medulloblastoma Cancer Stem Cells (SHH MB CSCs) vs. Neural Stem Cells (NSCs). The entire dataset is reported in Table S1; adj., adjusted.

miRNA	Log2 (Fold-Change)	adj. *p*-Value
mmu-miR-466i-5p	5.49	3.90 × 10^−3^
mmu-miR-615-3p	4.51	4.43 × 10^−2^
mmu-miR-871-3p	3.87	2.54 × 10^−4^
mmu-miR-708-3p	3.30	2.69 × 10^−2^
mmu-miR-135a-5p	2.90	4.10 × 10^−2^
mmu-miR-212-3p	2.83	4.19 × 10^−2^
mmu-miR-193a-5p	2.79	2.25 × 10^−2^
mmu-miR-330-5p	2.72	3.73 × 10^−2^
mmu-miR-212-5p	2.62	3.73 × 10^−2^
mmu-miR-193a-3p	2.50	8.88 × 10^−7^
mmu-miR-132-3p	2.49	4.43 × 10^−2^
mmu-miR-466h-3p	2.38	4.92 × 10^−2^
mmu-miR-150-5p	2.17	2.16 × 10^−2^
mmu-miR-139-5p	2.14	4.18 × 10^−2^
mmu-miR-195a-5p	2.11	5.53 × 10^−4^
mmu-miR-145a-5p	2.05	4.43 × 10^−2^
mmu-miR-146a-5p	2.03	3.26 × 10^−3^
mmu-miR-92a-1-5p	1.94	5.98 × 10^−3^
mmu-miR-23b-3p	1.84	3.43 × 10^−2^
mmu-miR-365-2-5p	1.84	3.20 × 10^−2^

**Table 2:** Top 20 down-regulated miRNAs in SHH MB CSCs vs. NSCs. The entire dataset is reported in Table S2; adj., adjusted.

miRNA	log2 (Fold-Change)	adj. *p*-Value
mmu-miR-8102	−11.57	2.85 × 10^−26^
mmu-miR-28c	−10.88	5.04 × 10^−31^
mmu-miR-486-3p	−9.67	1.56 × 10^−20^
mmu-miR-34a-5p	−8.66	3.04 × 10^−44^
mmu-miR-3084-5p	−8.48	3.90 × 10^−20^
mmu-miR-3080-3p	−7.77	1.87 × 10^−9^
mmu-miR-6922-5p	−7.44	2.27 × 10^−12^
mmu-miR-5122	−7.16	6.19 × 10^−9^
mmu-miR-31-5p	−7.02	1.68 × 10^−10^
mmu-miR-219c-5p	−6.93	1.00 × 10^−17^
mmu-miR-6944-5p	−6.91	1.11 × 10^−8^
mmu-miR-106a-5p	−6.79	9.35 × 10^−9^
mmu-let-7k	−6.78	9.79 × 10^−12^
mmu-miR-3074-5p	−6.40	2.11 × 10^−12^
mmu-miR-7036b-5p	−6.37	1.98 × 10^−8^
mmu-miR-7081-3p	−6.29	6.37 × 10^−14^
mmu-miR-6936-3p	−6.07	1.11 × 10^−8^
mmu-miR-7066-5p	−6.03	2.92 × 10^−6^
mmu-miR-378b	−6.01	1.37 × 10^−10^
mmu-miR-5099	−5.99	9.79 × 10^−12^

**Table 3:** Top 20 KEGG (Kyoto Encyclopedia of Genes and Genomes) pathways from enrichment analysis of SHH MB CSC transcriptome. The entire table is reported in Table S4.

#Pathway ID	Pathway Description	Observed Gene Count	False Discovery Rate
1100	Metabolic pathways	216	1.90 × 10^−27^
5016	Huntington’s disease	92	2.54 × 10^−51^
3010	Ribosome	91	3.04 × 10^−69^
190	Oxidative phosphorylation	83	6.82 × 10^−56^
5012	Parkinson’s disease	81	6.49 × 10^−50^
5010	Alzheimer’s disease	79	9.89 × 10^−41^
4932	Non-alcoholic fatty liver disease (NAFLD)	65	2.16 × 10^−30^
4141	Protein processing in endoplasmic reticulum	64	2.92 × 10^−26^
3040	Spliceosome	63	9.69 × 10^−34^
5169	Epstein–Barr virus infection	54	2.04 × 10^−14^
3013	RNA transport	52	1.98 × 10^−17^
4151	PI3K-Akt signaling pathway	48	7.44 × 10^−4^
1120	Microbial metabolism in diverse environments	47	5.73 × 10^−13^
5200	Pathways in cancer	46	5.82 × 10^−4^
1200	Carbon metabolism	44	2.69 × 10^−19^
4110	Cell cycle	44	6.66 × 10^−17^
4120	Ubiquitin mediated proteolysis	40	4.50 × 10^−12^
5203	Viral carcinogenesis	40	6.48 × 10^−7^
4810	Regulation of actin cytoskeleton	40	5.49 × 10^−6^
5205	Proteoglycans in cancer	40	1.07 × 10^−5^

**Table 4:** Top 20 KEGG pathways from enrichment analysis of up-regulated miRNAs in SHH MB CSC. The entire table is reported in Table S5.

KEGG Pathway	*p*-Value	#Genes	#miRNAs
Pathways in cancer	4.18 × 10^−8^	152	31
PI3K-Akt signaling pathway	1.28 × 10^−5^	130	30
MAPK signaling pathway	5.32 × 10^−6^	100	31
Focal adhesion	1.59 × 10^−6^	91	29
Proteoglycans in cancer	4.18 × 10^−8^	90	28
cAMP signaling pathway	3.92 × 10^−6^	86	29
Regulation of actin cytoskeleton	5.54 × 10^−5^	84	30
Hippo signaling pathway	3.92 × 10^−6^	68	28
Transcriptional misregulation in cancer	4.80 × 10^−5^	67	27
Axon guidance	2.75 × 10^−7^	66	26
Protein processing in endoplasmic reticulum	1.26 × 10^−4^	66	27
FoxO signaling pathway	3.92 × 10^−6^	63	26
Wnt signaling pathway	3.92 × 10^−6^	63	27
Signaling pathways regulating pluripotency of stem cells	8.71 × 10^−7^	62	30
Thyroid hormone signaling pathway	4.53 × 10^−7^	53	27
Choline metabolism in cancer	8.79 × 10^−5^	48	28
Prostate cancer	6.35 × 10^−6^	45	27
ErbB signaling pathway	9.09 × 10^−6^	45	27
Phosphatidylinositol signaling system	2.15 × 10^−6^	38	23
Renal cell carcinoma	4.70 × 10^−6^	35	27

**Table 5:** Top 20 KEGG pathways from enrichment analysis of down-regulated miRNAs in SHH MB CSC. The entire table is reported in Table S6.

KEGG Pathway	*p*-Value	#Genes	#miRNAs
Pathways in cancer	8.24 × 10^−7^	209	72
PI3K-Akt signaling pathway	3.39 × 10^−5^	182	70
MAPK signaling pathway	1.41 × 10^−6^	141	65
Endocytosis	8.24 × 10^−7^	123	60
Regulation of actin cytoskeleton	5.28 × 10^−6^	123	62
Focal adhesion	1.45 × 10^−5^	117	62
Rap1 signaling pathway	2.80 × 10^−5^	116	65
Proteoglycans in cancer	8.24 × 10^−7^	114	68
cAMP signaling pathway	4.14 × 10^−4^	106	69
Hippo signaling pathway	7.77 × 10^−8^	89	56
Axon guidance	5.69 × 10^−8^	83	54
FoxO signaling pathway	6.17 × 10^−6^	82	56
Thyroid hormone signaling pathway	7.84 × 10^−8^	67	50
TGF-beta signaling pathway	4.67 × 10^−4^	50	48
ECM-receptor interaction	4.12 × 10^−11^	48	45
Adherens junction	3.60 × 10^−8^	48	48
GABAergic synapse	4.93 × 10^−8^	48	47
Arrhythmogenic right ventricular cardiomyopathy (ARVC)	3.29 × 10^−7^	46	45
Amphetamine addiction	3.32 × 10^−5^	41	47
Long-term depression	6.80 × 10^−6^	39	49

**Table 6:** Overall survival of SHH MB patients (GEO ID: gse85217); *df*, degrees of freedom.

**Infant and Pediatric SHH MB Patients (Infant: 0–3 Years *n* = 50, Pediatric: 4–10 Years *n* = 45; 10–17 Years *n* = 26; Total *n* = 121), *df* = 1, chi, chi Squared Test.**					
**Gene**	**Affymetrix Probe**	**Bonferroni Adjusted *p*-Values**	**chi**	**Raw *p*-Value**	**Worse Overall Survival with**
CHUK	7935707	2.50 × 10^−2^	13.54	2.30 × 10^−4^	Low expression
CTNNB1	8079021	1.90 × 10^−5^	27.27	1.80 × 10^−7^	Low expression
DDIT4	7928308	2.00 × 10^−2^	13.93	1.90 × 10^−4^	Low expression
RHEB	8143957	2.00 × 10^−3^	18.31	1.90 × 10^−5^	High expression
UBQLN1	8161988	1.90 × 10^−2^	14.05	1.80 × 10^−4^	High expression
**Infant SHH MB Patients (Infant: 0–3 Years *n* = 50), *df* = 1**					
**Gene**	**Affymetrix Probe**	**Bonferroni-Adjusted *p*-Values**	**chi**	**Raw *p*-Value**	**Worse Overall Survival with**
MBTPS1	8003089	4.90 × 10^−2^	10.20	1.40 × 10^−3^	High expression
RAF1	8085374	4.70 × 10^−2^	10.27	1.40 × 10^−3^	High expression
SAR1B	8114193	3.30 × 10^−4^	19.64	9.40 × 10^−6^	Low expression
TXNDC5	8123802	1.80 × 10^−2^	12.10	5.00 × 10^−4^	High expression
UBE4B	7897527	6.20 × 10^−3^	14.05	1.80 × 10^−4^	Low expression
VCP	8160914	4.20 × 10^−2^	10.47	1.20 × 10^−3^	High expression
**Pediatric SHH MB Patients (Pediatric: 4–10 Years *n* = 45; 10–17 Years *n* = 26, Total *n* = 71), *df* = 1, chi, chi Squared Test.**					
**Gene**	**Affymetrix Probe**	**Bonferroni-Adjusted *p*-Values**	**chi**	**Raw *p*-Value**	**Worse Overall Survival with**
CHUK	7935707	3.20 × 10^−2^	11.87	5.70 × 10^−4^	Low expression
CTNNB1	8079021	1.40 × 10^−6^	31.02	2.50 × 10^−8^	Low expression
PPP2R1A	8030881	2.40 × 10^−2^	12.39	4.30 × 10^−4^	High expression
RAD23B	8157125	2.40 × 10^−4^	21.17	4.20 × 10^−6^	High expression
RHEB	8143957	3.10 × 10^−2^	11.93	5.50 × 10^−4^	High expression
UBQLN1	8161988	4.70 × 10^−5^	24.27	8.40 × 10^−7^	High expression
**Adult SHH MB Patients (Adult: >18 years, *n* = 49), *df* = 1, chi, chi Squared Test.**					
**Gene**	**Affymetrix Probe**	**Bonferroni-Adjusted *p*-Values**	**chi**	**Raw *p*-Value**	**Worse Overall Survival with**
EGFR	8132860	4.20 × 10^−2^	10.45	1.20 × 10^−3^	Low expression
MAPK3	8000811	3.90 × 10^−2^	10.59	1.10 × 10^−3^	High expression

**Table 7:** Overall survival of SHH MB patients, divided in the four SHH subtypes (GEO ID: gse85217).

**SHHα MB Pediatric Patients (*n* = 41), *df* = 1, chi, chi Squared Test.**					
**Gene**	**Affymetrix Probe**	**Bonferroni-Adjusted *p*-Values**	**chi**	**Raw *p*-Value**	**Worse Overall Survival with**
CTNNB1	8079021	8.90 × 10^−3^	12.82	3.40 × 10^−4^	Low expression
PDIA3	7983274	2.50 × 10^−2^	10.93	9.40 × 10^−4^	Low expression
RAD23B	8157125	7.30 × 10^−5^	21.95	2.80 × 10^−6^	High expression
RAF1	8085374	1.70 × 10^−2^	11.60	6.60 × 10^−4^	Low expression
RHEB	8143957	4.10 × 10^−2^	9.97	1.60 × 10^−3^	High expression
UBQLN1	8161988	3.80 × 10^−5^	23.18	1.50 × 10^−6^	High expression
YWHAH	8072577	1.50 × 10^−2^	11.88	5.70 × 10^−4^	High expression
**SHHβ MB Infant Patients (*n* = 21), *df* = 1, chi, chi Squared Test.**					
**Gene**	**Affymetrix Probe**	**Bonferroni-Adjusted *p*-Values**	**chi**	**Raw *p*-Value**	**Worse Overall Survival with**
UBE4B	7897527	4.60 × 10^−2^	7.11	7.70 × 10^−3^	Low expression
**SHHγ MB Infant Patients (*n* = 25)**					
**Gene**	**Affymetrix Probe**	**Bonferroni-Adjusted *p*-Values**	**chi**	**Raw *p*-Value**	**Worse Overall Survival with**
**SHHδ MB Adult Patients (*n* = 41), *df* = 1, chi, chi Squared Test.**					
Gene	**Affymetrix Probe**	**Bonferroni-Adjusted** ***p*-Values**	**chi**	**Raw *p*-Value**	**Worse Overall Survival with**
CHUK	7935707	1.20 × 10^−2^	12.20	4.80 × 10^−4^	Low expression
DNAJB11	8084634	6.00 × 10^−3^	13.57	2.30 × 10^−4^	Low expression

**Table 8:** Comparison of expression levels of genes of interest (Table 6) in WNT, SHH, Group 3, and Group 4 subtypes (GEO ID: gse85217).

**Infant and Pediatric MB Patients (SHH *n* = 146, WNT *n* = 51, Group 3 *n* = 131, Group 4 *n* = 300)**						
**Gene**	**Affymetrix Probe**	**Expression**	**Group 3 vs. SHH *p*-Value**	**Group 4 vs. SHH *p*-Value**	**WNT vs. SHH *p*-Value**	**Worse Overall Survival with**
CHUK	7935707	Lowest in SHH	5.70 × 10^−15^	5.00 × 10^−13^	2.30 × 10^−4^	Low expression
CTNNB1	8079021	Highest in SHH	1.70 × 10^−24^	1.60 × 10^−36^	1.50 × 10^−9^	Low expression
DDIT4	7928308	Highest in SHH	1.10 × 10^−6^	3.90 × 10^−44^	7.80 × 10^−6^	Low expression
RHEB	8143957	Highest in Group 4	0.87	1.00 × 10^−2^	9.90 × 10^−4^	High expression
UBQLN1	8161988	Highest in Group 3 and Group 4	3.40 × 10^−6^	2.00 × 10^−16^	0.15	High expression
**Infant MB patients (SHH *n* = 62, WNT *n* = 1, Group 3 *n* = 24, Group 4 *n* = 11)**						
**Gene**	**Affymetrix Probe**	**Expression**	**Group 3 vs. SHH *p*-Value**	**Group 4 vs. SHH *p*-Value**	**WNT vs. SHH *p*-Value**	**Worse Overall Survival with**
MBTPS1	8003089	Lowest in SHH	9.10 × 10^−4^	1.60 × 10^−4^	-	High expression
RAF1	8085374	-	0.21	0.34	-	High expression
SAR1B	8114193	Lowest in SHH	2.20 × 10^−9^	1.10 × 10^−4^	-	Low expression
TXNDC5	8123802	-	0.01	0.31	-	High expression
UBE4B	7897527	Highest in Group 3 and Group 4	0.27	1.80 × 10^−3^	-	Low expression
VCP	8160914	-	0.32	0.07	-	High expression
**Pediatric MB patients (SHH *n* = 84, WNT *n* = 50, Group 3 *n* = 107, Group 4 *n* = 289)**						
**Gene**	**Affymetrix Probe**	**Expression**	**Group 3 vs. SHH *p*-Value**	**Group 4 vs. SHH *p*-Value**	**WNT vs. SHH *p*-Value**	**Worse Overall Survival with**
CHUK	7935707	Lowest in SHH	5.80 × 10^−15^	1.20 × 10^−14^	3.00 × 10^−6^	Low expression
CTNNB1	8079021	Highest in SHH	3.50 × 10^−14^	5.90 × 10^−20^	8.10 × 10^−6^	Low expression
PPP2R1A	8030881	-	0.09	0.02	0.49	High expression
RAD23B	8157125	Lowest in SHH	4.20 × 10^−13^	9.10 × 10^−17^	1.80 × 10^−7^	High expression
RHEB	8143957	Lowest in WNT	0.87	5.30 × 10^−12^	2.10 × 10^−4^	High expression
UBQLN1	8161988	Lowest in SHH	1.40 × 10^−8^	9.10 × 10^−23^	5.20 × 10^−3^	High expression
**Adult MB patients (SHH *n* = 69, WNT *n* = 13, Group 3 *n* = 5, Group 4 *n* = 14)**						
**Gene**	**Affymetrix Probe**	**Expression**	**Group 3 vs. SHH *p*-Value**	**Group 4 vs. SHH *p*-Value**	**WNT vs. SHH *p*-Value**	**Worse Overall Survival with**
EGFR	8132860	Highest in SHH	1.20 × 10^−3^	2.60 × 10^−4^	5.00 × 10^−4^	Low expression
MAPK3	8000811	Lowest in WNT and Group 3	7.10 × 10^−3^	0.6	0.03	High expression

## Supplementary Materials

Supplementary materials can be found at http://www.mdpi.com/1422-0067/19/8/2326/s1.
